# Metformin: A Potential Therapeutic Tool for Rheumatologists

**DOI:** 10.3390/ph13090234

**Published:** 2020-09-04

**Authors:** Teresa Salvatore, Pia Clara Pafundi, Raffaele Galiero, Klodian Gjeloshi, Francesco Masini, Carlo Acierno, Anna Di Martino, Gaetana Albanese, Maria Alfano, Luca Rinaldi, Ferdinando Carlo Sasso

**Affiliations:** 1 Department of Precision Medicine, University of Campania “Luigi Vanvitelli”, Via de Crecchio, 7, I-80138 Naples, Italy; teresa.salvatore@unicampania.it; 2 Department of Advanced Medical and Surgical Sciences, University of Campania “Luigi Vanvitelli”, Piazza L. Miraglia, 2, I-80138 Naples, Italy; piaclara.pafundi@unicampania.it (P.C.P.); raffaele_ga@outlook.it (R.G.); klodian87@yahoo.it (K.G.); masini.fr@gmail.com (F.M.); carlo894@gmail.com (C.A.); annadimarti@alice.it (A.D.M.); gaetanaalbanese@hotmail.it (G.A.); maria.alfano2@libero.it (M.A.); luca.rinaldi@unicampania.it (L.R.)

**Keywords:** metformin, type 2 diabetes, rheumatology, inflammation, autoimmune diseases

## Abstract

Metformin is an oral antihyperglycemic drug widely used to treat type 2 diabetes, acting via indirect activation of 5′ Adenosine Monophosphate-activated Protein Kinase (AMPK). Actually, evidence has accumulated of an intriguing anti-inflammatory activity, mainly mediated by AMPK through a variety of mechanisms such as the inhibition of cytokine-stimulated Nuclear Factor-κB (NF-κB) and the downregulation of the Janus Kinase/Signal Transducer and Activator of Transcription (JAK/STAT) signaling pathways. Moreover, AMPK plays an important role in the modulation of T lymphocytes and other pivotal cells of the innate immune system. The current understanding of these AMPK effects provides a strong rationale for metformin repurposing in the management of autoimmune and inflammatory conditions. Several studies demonstrated metformin’s beneficial effects on both animal and human rheumatologic diseases, especially on rheumatoid arthritis. Unfortunately, even though data are large and remarkable, they almost exclusively come from experimental investigations with only a few from clinical trials. The lack of support from prospective placebo-controlled trials does not allow metformin to enter the therapeutic repertoire of rheumatologists. However, a large proportion of rheumatologic patients can currently benefit from metformin, such as those with concomitant obesity and type 2 diabetes, two conditions strongly associated with rheumatoid arthritis, osteoarthritis, and gout, as well as those with diabetes secondary to steroid therapy.

## 1. Introduction

Metformin is an oral antihyperglycemic drug widely used in type 2 diabetes (T2DM) treatment. Most of its metabolic effects are exerted via a direct inhibition of the mitochondrial respiratory chain complex I, which results in Adenosine Tri-Phosphate (ATP) depletion and Adenosine Mono-Phosphate (AMP) increase [1]. As a consequence, the phosphorylated activation of the 5′ adenosine monophosphate-activated protein kinase (AMPK) is indirectly induced [2].

AMPK is a key regulator of metabolism which senses increases in the intracellular ratio of AMP and/or Adenosine Di-Phosphate (ADP) to ATP following cellular stress (i.e., nutrient deficiency, hypoxia, and inflammation), and which subsequently triggers a metabolic switch from ATP consumption to ATP generation in order to maintain energy homeostasis [3].

Actually, evidence has accumulated of the pleiotropic effects of metformin, including an intriguing anti-inflammatory activity. The improvement in hyperglycemia, insulin resistance, and lipid profile due to this drug may, on its own, mitigate the chronic inflammation [4]. A 10-years study on rat myocardium showed an increase of the natural antilysosomal action of Zn^2+^ by metformin. Such a chloroquine-mimetic effect was interpreted as the evidence of an intrinsic anti-inflammatory power of the drug [5]. Some anti-inflammatory effects may be explained by the biguanide’s favorable impact on human microbiota and, therefore, on immune system [6]. However, the main mediator of metformin anti-inflammatory properties seems to be AMPK activation, more likely linked with the control of inflammation and immunity via a variety of mechanisms [7,8].

## 2. Anti-Inflammatory and Immuno-Modulating Effects of AMPK

**Figure 1:**
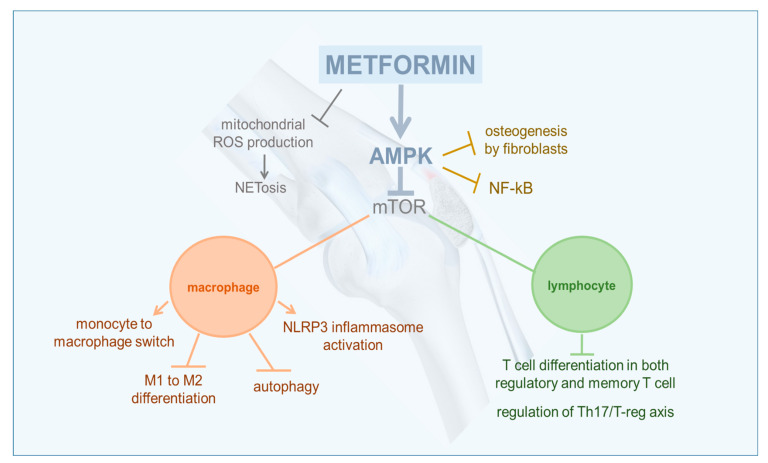
Simplified scheme of anti-inflammatory and immunomodulating effects of metformin.

Activated AMPK represents a key control point of a series of inter-connected inflammatory signaling pathways (Figure 1).

AMPK activation is associated with the inhibition of cytokine-stimulated nuclear factor-κB (NF-κB), a critical pathway for proinflammatory effects mediated by tumor necrosis factor (TNF)-α, as well as with the downregulation of the Janus kinase/signal transducer and activator of transcription (JAK/STAT) signaling pathways, a crucial driver of cytokine signaling, cell growth, and apoptosis [7,9]. In addition, the transcriptional factor STAT3 (one among the seven STAT proteins) was demonstrated to be involved in the differentiation of T helper 17 (Th17), follicular T helper (Tfh), and B cells [10,11,12].

The mammalian target of rapamycin (mTOR) is a downstream molecule in the AMPK pathway tightly associated with both glucose and lipid metabolism regulation, as well as with cell growth, proliferation, and survival, by sensing signals from growth factors, cytokines, and metabolic status [13]. As an example, the mTOR pathway stimulates T cells to differentiate into effector T cells, whereas mTOR-deficient T cells differentiate into regulatory T (Treg) cells [14]. Increased mTOR signaling is involved in multiple pathological conditions [15]. Moreover, rapamycin, the most potent mTOR inhibitor, was suggested as an add-on therapy in rheumatoid arthritis (RA) and systemic lupus erythematosus (SLE) [16,17]. Even metformin was proven to be able to significantly inhibit mTOR phosphorylation through AMPK activation [18].

As aforementioned, AMPK plays an important role in the modulation of T-lymphocyte proliferation and differentiation, but this enzyme complex critically contributes to shaping the activity of other pivotal cells of the innate immune system such as macrophages, neutrophils, and dendritic cells [19].

On the whole, the current understanding of the anti-inflammatory effects of AMPK provides a strong rationale for the development of novel AMPK activators, even for the repurposing of those already clinically available. Here, we describe the available preclinical and clinical data supporting metformin, an indirect activator of AMPK, as a potential therapeutic strategy for the management of a range of rheumatologic autoimmune and inflammatory conditions (Table 1).

## 3. Rheumatoid Arthritis

Rheumatoid arthritis (RA) is a common autoimmune disease mainly associated with chronic, painful arthritis. Affected joints are characterized by abundant cellular infiltration of the synovial membrane, from which multiple inflammatory cytokines and matrix-degrading enzymes are released, which triggers the progressive destruction of adjacent cartilage and bone [44].

In the pre-methotrexate era, phenformin, an old biguanide withdrawn worldwide from the market due to safety issues, was tested with some success for RA treatment due to its “fibrinolytic” activity. An improvement of clinical disease and decreased erythrocyte sedimentation rate levels were shown [45].

AMPK activation may control inflammatory arthritis. Such an assertion is supported by the mildly increased clinical serum-induced arthritis observed in AMPKα1-deficient mice as compared to wild-type controls [46]. In the same study, AMPK activation by specific AMPK agonist A-769662 suppressed inflammatory arthritis in murine models of antigen-induced arthritis (AIA) and passive K/BxN serum-induced arthritis, suggesting that targeted AMPK activation may represent an effective therapeutic strategy for RA.

Several experimental investigations on RA murine models focused on the therapeutic role of metformin in autoimmune arthritis. Proinflammatory cytokines are intimately involved in RA pathogenesis. In particular, interleukin IL-17 produced by Th17 cells plays a key role in joint inflammation and destruction due to its powerful inflammatory properties [47], and its ability to induce both matrix metalloproteinases and osteoclasts [48,49]. Even though Th17 cells were identified and named due to their production of IL-17, they also produce a series of additional effector molecules, such as IL-22, IL-26, interferon gamma, TNF-α, granulocyte-macrophage colony-stimulating factor, and C–C motif chemokine ligand 20, which may contribute to the pathology of RA [50]. AMPK is associated with Th17 cell suppression, thus suggesting a potential therapeutic role of AMPK agonists in RA.

The anti-inflammatory effect of metformin was first investigated in a well-established mouse model of autoimmune arthritis (collagen antibody-induced arthritis (CAIA)) [20]. It was demonstrated that the drug lowered in vivo the increase of Th17 cells with lower production of proinflammatory cytokines and suppressed in ex vivo Th17 differentiation by inhibiting STAT3 phosphorylation via the AMPK/mTOR pathway. Furthermore, a significant improvement in arthritis score with a dose-dependent reduction in inflammatory cell infiltration was also observed. Based on these findings, the authors suggested the therapeutic value of metformin in RA treatment.

The Treg cell regulatory function is known to be deranged in RA patients [21]. A later study on a murine model of collagen-induced arthritis (CIA) demonstrated that metformin tempered arthritis severity by suppressing Th17 cells and simultaneously enhancing Treg cells [22]. Hence, AMPK activation and mTOR inhibition would represent an efficient way to regulate the Th17/Treg axis. Metformin also suppressed osteoclast differentiation both in vivo and in vitro, consistently with the reported negative regulation of osteoclastogenesis by the AMPK-mediated inhibition of mTOR [23].

RA pathogenesis also entails a dysregulation of autophagic pathways, notably involved in the innate and adaptive immune response by recycling and removing harmful protein aggregates and damaged cell organelles [51]. AMPK activation and mTOR negative regulation are both implicated in the autophagy signaling network [52], a central regulator in both inducing and keeping inflammation [53]. The role of autophagy was investigated in an experimental model of arthritis sharing many features with human RA, induced by the passive transfer of arthrogenic autoantibodies from KRN to wild mice [24]. In this model, autophagy was initiated but the autophagic flux was severely impaired. Such a defect was corrected by metformin treatment via stimulation of macrophage AMPK activity and mTOR signaling interruption. In vivo, metformin treatment significantly suppressed clinical arthritis and inflammatory cytokine production. In vitro, the drug suppressed, in a dose-dependent manner, the release of TNF-α, interleukin IL-6, and MCP-1 by macrophages, while enhancing IL-10 release [54].

It was reported that metformin is able to decrease mitochondrial activity and suppress respiration at complex I in peripheral blood mononuclear cells and platelets of healthy subjects at concentrations clinically relevant for intoxication, determining the increased production of lactate [55]. Indeed, several data confirm the beneficial mitochondrial effects of metformin, exerted by diminishing Reactive Oxygen Species (ROS) overproduction and oxidative stress [56]. On the other hand, it was observed that CoQ10 can attenuate mitochondrial dysfunction and promote mitochondrial activity [57]. As mitochondrial dysfunction is involved in the inflammatory response, the effects of combined therapy with metformin and CoQ10 were investigated in a CIA murine model [25]. The combined therapy displayed an additive effect, with a higher improvement of both mitochondrial function and arthritis as compared to either metformin or CoQ10 alone. The anti-inflammatory response was associated with inhibition of IL-17 expression, reduction of Th17 cells, and Treg cell induction. Moreover, a reduced osteoclastogenesis, established by a lowered number of Tartrate resistant acid phosphatase (TRAP)-positive multinucleated cells, was reported in in vitro tests. Due to the involvement of both metformin [22] and CoQ10 [58] in the inhibition of osteoclastogenesis, and to the exacerbating role of osteoclasts in joint inflammation and bone destruction [59], the authors concluded that combined therapy may exert a better preventive role than single drugs in autoimmune arthritis.

Obesity is defined as a chronic low-grade inflammatory disease with an increase in the expression of proinflammatory cytokines and a concomitant decrease in the expression of anti-inflammatory ones [60]. The therapeutic effect and the underlying mechanisms of metformin action were investigated in a CIA murine model with high-fat diet-induced obesity [26]. As a result, metformin dampened CIA development in obese mice. This antiarthritic effect depended on restoration of the reciprocal Th17/Treg balance and correction of the metabolic dysfunction by upregulating BAT differentiation.

In a model of high-fat diet-fed obese C57/6J male mice, metformin treatment reduced IL-6 and TNF-α serum levels and modulated the AMPK-mediated macrophage polarization toward the anti-inflammatory M2 phenotype [27]. Due to the abundance of inflammatory M1 macrophages in the synovial tissue in active RA [61], it was hypothesized that the favorable effects on these cells may contribute to the improvement of obesity-associated arthritis by metformin.

Evidence is growing with regard to the significant contribution of fibroblast-like synoviocytes (FLSs) to both the onset and the progression of RA by producing cytokines which prolong inflammation, as well as proteases contributing to the cartilage destruction [62]. A recent study on synovial tissue from RA patients during surgically treated knee arthroplasty demonstrated that metformin could inhibit RA-FLS proliferation in a dose- and time-dependent manner [28].

Some studies support the therapeutic potential of mTOR inhibitors in RA [63,64]. On the other hand, these have the intrinsic limitation of leading to mitochondrial dysfunction [65,66], a condition that exacerbates inflammation and oxidative stress and may contribute to the pathogenesis of various diseases, including RA [67]. To minimize mitochondrial dysfunction as a side effect of rapamycin, Kim et al. [29] assessed the therapeutic effect of a combined administration of rapamycin and metformin in a CIA obese mouse model. As a result, the combined therapy showed an increased therapeutic potential in arthritis improvement while not inducing mitochondrial dysfunction. The authors suggested that metformin in addition to rapamycin could represent a useful therapeutic option for RA obese patients.

Based on previous studies reporting an increased prevalence of altered glucose metabolism in RA patients [68,69], the relationship between insulin resistance and disease activity was recently further explored in an Irish cohort of 92 patients with RA [30]. Using ex vivo synovial explants from RA patients and osteoarthritis (OA) non-inflammatory controls, the activity of glucose transporters GLUT1 (largely insulin-dependent) and GLUT4 (activated under the influence of insulin) was measured. An increased GLUT1 and decreased GLUT4 expression in the lining layer of RA as compared to OA synovium was found. After metformin treatment, AMPK phosphorylation in RA synovial tissue appeared to increase, whilst a reduction of both GLUT1 expression and spontaneous IL-6, IL-8, and MCP-1 production was observed. These findings first provided direct evidence that insulin resistance may be a feature of chronic inflammation in synovial tissue beyond its known systemic effects. In addition, metformin may partly resolve synovial inflammation in RA.

As compared to the abundance of experimental studies on immune arthritis, clinical data on patients suffering from RA are still poor. A retrospective cohort study in Israel on patients initiated to metformin for T2DM between 1998 and 2014 reported a clinically important association between decreased risk of developing RA and higher exposure to metformin, intended as adherence to treatment and daily drug dosage [31]. An observational study on data from a Taiwan National Health Insurance Research Database found that patients with RA and T2DM under Cyclooxygenase (COX)-2 inhibitor and metformin had lower admission rates than those on COX-2 inhibitors alone [32]. Finally, a recent review strengthened metformin’s multiple benefits in RA patients not only in terms of joint involvement but also affecting other frequently associated illnesses such as cardiovascular disease and cancer [70].

## 4. Osteoarthritis

Osteoarthritis (OA) is the most common degenerative joint disease. Its hallmarks are progressive degeneration of articular cartilage, synovitis, subchondral bone sclerosis, and osteophyte formation.

An increasing body of evidence indicates that OA progression mainly depends on a chronic low-grade inflammation involving articular chondrocytes [71], and AMPK is a main actor in this inflammatory process [72]. AMPK deficiency in chondrocytes seems to accelerate the progression of both injury-induced and age-related OA in adult mice [73]. A decreased phosphorylation in the catalytic α subunit of AMPK was observed in mouse OA models and the knee cartilage of human OA [74,75]. AMPK activation by specific AMPK agonist A-769662 suppressed inflammatory arthritis in murine models of antigen-induced arthritis (AIA) and passive K/BxN serum-induced arthritis, suggesting that targeted AMPK activation may be an effective therapeutic strategy for OA [46]. Data on this topic are poor and even controversial. However, a very recent investigation [33] demonstrated that metformin administration shortly after joint injury in an OA murine model of destabilization of the medial meniscus (DMM) limited OA development and delayed OA progression, even relieving pain sensitivity. In addition, these benefits were lost in an OA model of AMPK α1 knockout (KO) and DMM mice, thus suggesting that the chondroprotective effect of the drug was mediated by upregulating AMPK α1 expression. The study, extended to nonhuman primates, confirmed the chondroprotection by metformin in a partial medial meniscectomy model of OA. The practical application suggested by authors was to use metformin in young patients shortly after a joint injury.

Up to two-thirds of elderly obese population are affected by knee OA [76], and over 50% of knee OA patients are obese [77]. As reported by a recent systematic review, even T2DM seems to be a risk factor for knee OA in dependence of obesity [78]. In addition to weight-related mechanical factors, this strong association is more likely supported by the obesity-associated promotion of systemic inflammation, oxidative stress, and accumulation of advanced glycation end products (AGEs) [79,80]. In particular, the metabolic perturbations typical of central obesity and metabolic syndrome (i.e., intracellular accumulation of succinate and citrate, increase in free fatty acids, hyperglycemia-induced AGEs) are important drivers of proinflammatory macrophage polarization and activity within synovial and adipose tissue, via alterations of AMPK and mTORC1, as well as of adipokine levels. This detrimental metabolic pattern also affects the cartilage through direct effects on chondrocytes [81]. Given the biological effects of metformin targeting the underlying OA pathogenic mechanisms, the drug might be considered a potential disease-modifying agent in the obese phenotype with knee OA, in addition to weight loss. Some data on humans are available. A recent prospective cohort study reported a relationship between metformin use and reduced knee OA progression in obese individuals, established by a decreased rate of medial cartilage volume loss over four years and a trend toward a decreased risk of total knee replacement over six years in drug users as compared to non-users [34].

In a population of Hispanics from Puerto Rico, DM patients were more likely to have hand or knee OA than nondiabetic subjects [82]. Moreover, a meta-analysis also showed a higher prevalence of OA in DM patients [83]. Conversely, subjects with OA had an increased risk of developing T2DM as compared to their age-sex matched non-OA counterparts [84]. Knowledge about this diabetes–arthritis connection is limited. A retrospective cohort study on a large sample size from a primary care population database in the United Kingdom (UK) failed to demonstrate a protective effect of metformin prescription on OA in T2DM patients [85]. On the contrary, a nationwide, retrospective, matched-cohort study in Taiwan found that the combination of metformin with COX-2 inhibitors in OA diabetic patients was associated with a decreased risk of joint replacement surgery by 25% over 10 years when compared with COX-2 inhibitors alone [35].

In vivo animal studies showed that pharmacological activation or genetic regulation of AMPK had preventive, curative, and potential reversal effects on pain in various models of nerve injury [86]. Thus, it could be hypothesized that metformin may be able to improve OA-related pain, as confirmed by a recent study on a destabilized medial meniscus (DMM) OA mouse model [36], in which both intragastric and intra-articular metformin attenuated articular cartilage degradation and modulated pain-related behavior, more likely through AMPK activation.

Multipotent mesenchymal stem cells (MSCs) present in various human tissues are able to differentiate into osteoblasts, chondrocytes, and adipocytes. They can protect against cartilage breakdown, as recently found in a murine model of OA [37]. In this study, intravenous administration of metformin-stimulated adipose tissue-derived human MSCs (Ad-hMSCs) showed superior chondroprotective and antinociceptive effects as compared to the untreated Ad-hMSCs. The authors suggested that the immune-regulatory property of metformin may represent a simple and innovative strategy to optimize MSCs as a type of cell therapy for OA.

## 5. Gout

Gout is a common inflammatory arthritis caused by deposition of monosodium urate (MSU) crystals in the joints. The major driver of inflammation is NLR family pyrin domain containing 3 (NLRP3) inflammasome activation, a process initiated when MSU crystals are taken up by resident macrophages. The inflammasome-mediated IL-1β release triggers an important inflammatory response, with vasodilatation and a rapid increase of neutrophil influx to the site of crystal deposition. Inflammation is amplified by recruited neutrophils whose responses to MSU crystals represent a key component of gout attacks [87]. It was observed that MSU priming in monocytes leads to mTOR activation in concert with IL-1β expression [88], and that mTOR inhibition with rapamycin in macrophages leads to proIL-1β degradation and lower NLRP3 inflammasome activation [89].

A recent study using a translational approach from ex vivo to in vitro and back to in vivo showed that ex vivo immune cells from gout patients exhibit higher relative gene expression of the mTOR pathway as compared to healthy controls, with monocytes being the most prominent mTOR expressers [38]. Using live imaging, it was observed that monocytes, on encountering MSU crystals, initiated cell death after contact and released a wide array of proinflammatory cytokines. By inhibiting mTOR signaling with either metformin or rapamycin, a reduction in the release of cell death and inflammatory mediators was obtained. Based on these findings, metformin may represent a suitable treatment for gout, even considering that biguanide seems to lower uric acid levels via a not clearly proven interference with the purine pathway [90].

In a large retrospective case–control study in T2DM patients, increased A1C levels but not use of antidiabetic drugs, lowered the risk of incident gout [91]. However, with respect to insulin and sulfonylureas, metformin appeared to lower the adjusted Odd Ratio (OR), even despite the lack of a consistent association with the duration of therapy. Two small-scale Russian studies on gout nondiabetic patients showed that metformin reduced the frequency of gout attacks and lowered circulating uric acid up to normouricemia in some cases [39].

As compared to the current drugs used in gout treatment, metformin has the potential advantage of targeting multiple aspects of the disease. That is, it inhibits inflammation, reduces hyperuricemia, and decreases the high cardiovascular and metabolic risk characteristic of gouty patients [92].

## 6. Systemic Lupus Erythematosus

Some findings indicate that metformin might be a promising approach to treat systemic lupus erythematosus (SLE).

The formation of neutrophil extracellular traps (NETs) is an ROS-dependent event that characterizes NETosis, a neutrophil-specific form of cell death different from apoptosis or necrosis [93]. NETs consist of DNA, histones, and antimicrobial peptides, and they are of major importance in SLE pathogenesis as they are a source of antiDNA autoantibody generation, while they activate plasmacytoid dendritic cells (PDCs) [94] and the type I interferon pathway [95].

Metformin is reported to exert a potent antioxidant activity mainly by enhancing the endogenous antioxidant enzymes and by downregulating Nicotinamide adenine dinucleotide phosphate (NADPH) oxidase, one of the major producers of cellular ROS enzymes, which is upregulated following exposure of cells to stress conditions (i.e., oxidative stress and inflammation) [56]. It follows, therefore, that metformin can also downregulate the NET pathway. It was reported, in an in vitro study, that metformin decreased NET DNA release in cultured neutrophils and inhibited IFNα generation by stimulated PDCs [40]. Based on these findings, 113 patients with mild or moderate SLE were enrolled in a randomized, open-label, proof-of-concept trial to evaluate the efficacy and safety of metformin as an adjunct treatment to corticosteroids and conventional immunosuppressive agents [40]. A 51% reduction in disease flare frequency in the metformin add-on group emerged, without risks for increased prednisone exposure.

It was also reported that Cluster of Differentiation 4 (CD4^+^) T cells from a lupus-prone mouse model, as well as from SLE patients, exhibited elevated glycolysis and mitochondrial oxidative metabolism as compared to nonautoimmune controls, both ex vivo and after in vitro activation [41]. Mouse treatment with a combination of metformin and 2DG, a glucose metabolism inhibitor, normalized T-cell metabolism restoring CD4^+^ T function and reversing disease phenotypes, including autoantibody production and renal disease.

## 7. Sjögren Syndrome

Few effective drugs are available against Sjögren syndrome (SS), an immune-related chronic inflammatory disease typically involving salivary and lacrimal glands. In SS, Th17 and Tfh cells are activated, and the balance with their regulatory counterparts (Treg and Tfr cells) may play a pathophysiological role [96].

The STAT3 pathway was found to be involved in SS [97]. Additionally, an mTOR-targeted drug strongly suppressed autoimmune dacryoadenitis in a mouse model of SS [98]. As a result, targeting STAT3/mTOR signaling with metformin could be effective against SS. Using an SS murine model, some investigators recently demonstrated that metformin suppressed effector T cells, induced regulatory T cells, and regulated B-cell differentiation [42]. In addition to histologic aspects, salivary gland inflammation and flow rate improved, thus establishing metformin as a potential therapy for SS.

## 8. Ankylosing Spondylitis

Ankylosing spondylitis (AS) is an autoimmune disease involving the axial skeleton, whose hallmark is pathological bone formation [99]. Even though the precise mechanisms underlying pathological osteogenesis remain poorly understood, fibroblasts seem to play an important role [100].

Metformin is suggested to have bone-protective properties [101,102], established by AMPK phosphorylation, and to alleviate the vascular calcification induced by vitamin D3 plus nicotine [103].

Metformin’s effects on fibroblasts harvested from capsular ligament of patients with femoral neck fracture and AS were recently investigated [43]. The results showed that osteogenic-specific markers were highly downregulated, and ossification was effectively inhibited after metformin addition. The levels of inflammation markers were also decreased. This potent anti-osteogenic effect on both AS fibroblasts and normal fibroblasts suggested metformin as potentially useful for AS treatment.

## 9. Conclusions

An increasing body of evidence shows that cellular stresses may affect immune and inflammatory status via AMPK activation, an event which triggers a complex set of protective mechanisms. The secondary anti-inflammatory/immunomodulating effects of the AMPK activator metformin may enable drug repurposing and expansion of available therapeutic options for a series of chronic rheumatologic disorders, particularly RA.

Metformin may be notably beneficial for long-term treatment regimens as it is an old and widely used drug, with two precious qualities: low cost and excellent safety profile. Metformin is not likely to cause hypoglycemia when used as a monotherapy, and common adverse effects are relatively mild and mainly represented by gastrointestinal intolerance. A more serious but very rare adverse effect is lactic acidosis, usually determined by drug misuse [104].

The data collected so far are large and considerable but unfortunately almost exclusively experimental. Until results are available from properly designed multicenter, randomized, placebo-controlled trials, metformin cannot enter the therapeutic repertoire proposed by the guidelines referred to by rheumatologists. As millions of people around the world use metformin, a valid and less expensive alternative could be to exploit epidemiological data extracted from the population of rheumatological patients with T2DM. At any rate, a large proportion of rheumatologic patients can currently benefit from the drug, e.g., those with concomitant obesity and T2DM, two conditions strongly associated with rheumatologic diseases such as AR, OA, and gout. In cases where secondary diabetes develops after steroid therapy, metformin can represent an optimal therapeutic option due to its glucose-lowering and anti-inflammatory properties. Nevertheless, metformin is well tolerated by patients without T2DM, e.g., women with polycystic ovary syndrome. Its use is even suggested by scientific societies in populations with metabolic syndrome and prediabetes. Moreover, the well-documented beneficial impact of metformin on cardiovascular disease and cancer [105] may be very useful when considering their close association with RA and gout [105,106,107,108].

In conclusion, the well-documented efficacy of metformin in improving phlogistic and immunological responses support its therapeutic potential as a promising pharmacological tool in rheumatologic diseases, most of all RA, although also OA, as there is currently no medication for the latter to either halt or decelerate clinical progression.

## Figures and Tables

**Table 1:** Experimental and clinical effects of metformin in rheumatologic autoimmune and inflammatory conditions.

**Rheumatoid arthritis (RA)**	Lower increase in Th17 cells with reduction of proinflammatory cytokines and improved arthritis score in a CAIA mouse model [20,21]
	Suppression of Th17 cells and enhancement of Treg cells in a CIA murine model [22]
	Suppression of osteoclast differentiation [23]
	Impaired autophagy correction with suppression of inflammatory cytokines and clinical arthritis in a murine model of immune arthritis [24]
	Higher reduction of Th17 cells, induction of Treg cells, and inhibition of osteoclastogenesis with higher arthritis improvement by metformin combined with CoQ10 vs. metformin or CoQ10 alone in a CIA murine model [25]
	Restoration of reciprocal Th17/Treg balance with dampened CIA development in a murine model of diet-induced obesity [26]
	Modulation of macrophage polarization toward M2 phenotype in a model of high-fat diet-fed obese C57/6J male mice [27]
	Inhibition of RA-FLS proliferation on synovial tissue from patients with RA [28]
	Mitochondrial dysfunction reduction by rapamycin combined with metformin vs. rapamycin alone in a CIA obese mouse model [29]
	Reduction of GLUT-1 expression in synovial tissue from patients with RA [30]
	Inverse association between risk of RA and exposure to metformin inT2DM patients [31]
	Lower admission rate of T2DM patients with RA treated with metformin and Cyclooxygenase (COX)-2 inhibitor vs. COX-2 inhibitors alone [32]
**Osteoarthritis (OA)**	Osteoarthritis limited development and delayed progression in a DMM murine model, not in an AMPK/α1 knockout DMM mice [33]
	Chondroprotection in a partial medial meniscectomy model of non-human primates [33]
	Reduced knee osteoarthritis progression in obese patients [34]
	Decreased risk of joint replacement surgery by 25% over 10 years [35]
	Improvement of osteoarthritis-related pain on a DMM OA mouse model [36]
	Chondroprotective and antinociceptive effect of intravenous [i.v.] administration of metformin-stimulated Ad-hMSCs [37]
**Gout**	Reduction in release of cell death and inflammatory mediators from monocytes encountering MSU crystals [38]
	Decrease of incident gout in T2DM patients and of gout attacks in gouty non-diabetic patients [39]
**Systemic lupus erythematous**	Reduction of NET DNA release in cultured neutrophils and inhibition of Interferon (INF)-α generation from stimulated PDCs [40]
	51% reduction of flares frequency in patients with mild or moderate disease [40]
	Restoration of Cluster of Differentiation (CD)4^+^ T function and reversion of disease phenotypes in a lupus-prone mouse model [41]
**Sjögren syndrome**	Suppression of effector T cells and induction of regulatory T cells in a murine model of Sjögren syndrome [42]
**Ankylosing spondylitis**	Potent antiosteogenic effect on human fibroblasts [43]

**Abbreviations:** AMPK, 5′ adenosine monophosphate-activated protein kinase; CAIA, collagen antibody-induced arthritis; CIA, collagen-induced arthritis; RA-FLS, rheumatoid arthritis fibroblast-like synoviocytes; GLUT1, glucose transporter 1; T2DM, type 2 diabetes mellitus; DMM, destabilization of medial meniscus; MSU, monosodium urate; NET, neutrophil extracellular trap; Ad-hMSCs, adipose tissue-derived human multipotent mesenchymal stem cells; PDCs, plasmacytoid dendritic cells.
